# GABA_B_ receptor‐mediated modulation of sensory neuron excitability: Roles of Ca_V_2.2, G‐protein‐coupled inwardly rectifying potassium (GIRK) channels, and hyperpolarisation‐activated cyclic nucleotide‐gated (HCN) channels in human and mouse nociception

**DOI:** 10.1113/EP093318

**Published:** 2025-11-30

**Authors:** Mariana Brizuela, Anuja R. Bony, Sonia Garcia‐Caraballo, David J. Adams, Stuart M. Brierley

**Affiliations:** ^1^ Visceral Pain Research Group South Australian Health and Medical Research Institute (SAHMRI) Adelaide South Australia Australia; ^2^ Faculty of Health and Medical Sciences University of Adelaide Adelaide South Australia Australia; ^3^ Molecular Horizons/Faculty of Science, Medicine and Health University of Wollongong Wollongong New South Wales Australia

**Keywords:** analgesic peptide, baclofen, colonic afferents, dorsal root ganglia, electrophysiology, ion channel, neuronal excitability

## Abstract

Chronic visceral pain is a key symptom of irritable bowel syndrome. Modulation of voltage‐gated calcium and potassium channels by G protein‐coupled receptors plays a key role in dampening nociceptive transmission. Both baclofen and the analgesic peptide α‐conotoxin Vc1.1 activate GABA_B_ receptors (GABA_B_R), resulting in inhibition of Ca_V_2.2 and Ca_V_2.3 calcium channels to reduce colonic nociception. Recent studies have also shown that GABA_B_R activation potentiates G‐protein‐coupled inwardly rectifying potassium (GIRK)‐1/2 channels in mammalian sensory afferent neurons. In this study, we investigated the expression of these ion channel targets in rodent and human dorsal root ganglion (DRG) neurons, including those innervating the colon. We examined how Ca_V_2.2 and GIRK channel antagonists, as well as a GIRK channel activator, influence the passive and active electrical properties of adult mouse DRG neurons. We also assessed the effects of α‐conotoxin Vc1.1 on neuronal excitability in the presence of the selective Ca_V_2.2 antagonist ω‐conotoxin CVIE and the GIRK channel activator ML297. We further evaluated the impact of the GIRK channel antagonist tertiapin‐Q on excitability in mouse colonic DRGs and afferents and explored the role of hyperpolarization‐activated cyclic nucleotide‐gated (HCN) channels. Our findings demonstrate that both Ca_V_2.2 inhibition and GIRK channel potentiation reduce excitability in mouse DRGs, likely mediating the antinociceptive effects of Vc1.1 and baclofen observed in vivo. However, GIRK channel potentiation appears to play only a limited role in modulating excitability in colon‐innervating DRGs and colonic afferents. These findings suggest that neurons innervating different body regions use distinct mechanisms to regulate excitability and nociceptive signalling.

## Abstract (highlights)

- **What is the central question of this study?**
- Do the ion channels modulated by α‐conotoxin Vc1.1, which influence excitability in somatic sensory neurons, also play comparable roles in visceral sensory neurons that innervate the colon?
- **What is the main finding and its importance?**
- Although Ca_V_2.2 inhibition and GIRK potentiation reduce excitability in somatic DRG neurons, GIRK activation exerts minimal effects in colon‐innervating DRG neurons. This highlights organ‐specific mechanisms of sensory regulation and may inform the development of more targeted therapies for visceral pain.

## 1 INTRODUCTION

Chronic pain is a major global health issue, affecting >30% of the population (Cohen et al., 2021). Chronic visceral pain is a particularly common form of chronic pain and is a key symptom of irritable bowel syndrome (IBS), a chronic gastrointestinal disorder affecting 7%–21% of individuals worldwide (Black & Ford, 2020; Camilleri, 2021). Although the pathophysiology of IBS is complex and not fully understood, substantial evidence indicates pain is driven by hypersensitivity of gut‐innervating afferents and dysfunction along the gut–brain axis (Brierley & Linden, 2014; Grundy et al., 2019). Peripheral hypersensitivity appears to be triggered by inflammatory or immune mediators, which contribute to the development of chronic visceral hypersensitivity (CVH) and neuroplastic changes within sensory pathways (Brierley & Linden, 2014; Grundy et al., 2019; Van Remoortel et al., 2024). These changes ultimately lead to heightened pain perception in the gastrointestinal tract (Brierley & Linden, 2014; Grundy et al., 2019). Upregulation of multiple ion channels and receptors in sensory afferents has been linked to the development of CVH in animal models (Cardoso et al., 2021; Castro et al., 2017, 2019; Desormeaux et al., 2018; Israel et al., 2019; Jiang et al., 2021; Osteen et al., 2016; Salvatierra et al., 2018). Consequently, targeting these ion channels and receptors may represent a promising therapeutic strategy for managing chronic visceral pain in IBS and chronic pain more broadly (Brizuela et al., 2021; Erickson et al., 2018; Grundy et al., 2019; Muttenthaler et al., 2021; Sadeghi et al., 2018b).

α‐Conotoxins are small, disulfide‐rich peptides – typically 12−30 amino acids in length – derived from the venom of marine cone snails. These peptides are promising drug candidates for the treatment of chronic pain, and chronic visceral pain, due to their ability to selectively target a wide range of membrane receptors and ion channels (Callaghan et al., 2008; Li et al., 2021; Schroeder & Craik, 2012). In particular, α‐conotoxin Vc1.1 has demonstrated anti‐nociceptive effects in vitro and anti‐hyperalgesic actions in various in vivo models of neuropathic pain (Clark et al., 2010; Klimis et al., 2011; Li et al., 2021). Historically, the analgesic effects of Vc1.1 were attributed to its inhibitory action on neuronal nicotinic acetylcholine receptors (nAChRs) (Frøsig‐Jørgensen et al., 2023; Livett et al., 2006). However, subsequent research has shown that its analgesic properties are primarily mediated through the activation of the G protein‐coupled γ‐aminobutyric acid type B receptor (GABA_B_R) (Bony et al., 2022; Callaghan et al., 2008; Frøsig‐Jørgensen et al., 2023; Sadeghi et al., 2017). GABA_B_Rs are broadly expressed on sensory neurons in both humans and rodents, including those innervating the gut (Castro et al., 2017). Our previous studies demonstrated that Vc1.1, along with its modified and cyclized form (cVc1.1), inhibits colonic sensory afferents, with these inhibitory effects being further amplified in an animal model of IBS (Carstens et al., 2016; Castro et al., 2018, 2017; Sadeghi et al., 2018a). Importantly, we identified that the mechanism of action of Vc1.1 involves the activation of GABA_B_Rs on colonic afferents, leading to downstream inhibition of voltage‐gated calcium channels Ca_V_2.2 and Ca_V_2.3 and a reduction in the excitability of dorsal root ganglion (DRG) neurons innervating the mouse colon (Castro et al., 2018, 2017). Furthermore, we have shown that native human DRG neurons and human pluripotent stem cell (hPSC)‐derived sensory neurons are inhibited by the GABA_B_R agonists, baclofen and Vc1.1 (Castro et al., 2017; St Clair‐Glover et al., 2025). Notably, the latter study confirmed the expression of G‐protein‐coupled inwardly rectifying potassium (GIRK) channels in hPSC‐derived sensory neurons. This is significant, as GABA_B_R activation is known to reduce neuronal excitability not only through inhibition of Ca_V_ channels, but also via potentiation of GIRK channels (Bony et al., 2022).

In this study, we investigated the role of GIRK channels in modulating neuronal excitability in mouse DRG neurons, including those specifically innervating the colon. We assessed the contributions of Ca_V_2.2 and GIRK channels to the passive and active electrical properties of adult mouse DRG neurons. Additionally, we examined whether inhibitory effects of Vc1.1 on neuronal excitability are mediated by GABA_B_R activation, leading to downstream modulation of Ca_V_2.2 and GIRK1/2 channels. Finally, we explored the roles of GIRK channels and hyperpolarization‐activated cyclic nucleotide‐gated (HCN) channels in regulating the excitability of colon‐innervating DRG neurons and colonic afferents, to better define the mechanisms by which α‐conotoxins such as Vc1.1 modulate colonic neuronal excitability.

## 2 METHODS

### 2.1 Ethical approval

All animal procedures were conducted in accordance with the University of Wollongong Animal Ethics Committee (AEC) guidelines and regulations (under AEC protocol reference numbers AE16/10 and AE16/10r19, approved August 2016) and SAHMRI's AEC (ethics approval number SAM 195, approved in January 2016). AEC guidelines comply with the ‘Australian code of practice for the care and use of animals for scientific purposes’, and the ARRIVE guidelines on reporting experiments involving animals (Percie du Sert et al., 2020).

For studies conducted at the University of Wollongong, adult male C57BL/6 mice (8–14 weeks old; RRID:MGI:6200612) were obtained from Australian Bioresources (Moss Vale, NSW, Australia) and housed in individually ventilated cages under a 12 h light–dark cycle with plastic shelters, nesting material, food pellets and water available ad libitum. For studies performed at SAHMRI, male C57BL/6J mice aged 13–17 weeks were used. These mice were acquired from an in‐house C57BL/6J breeding programme (strain no. 000664; originally purchased from The Jackson Laboratory, barn MP14) within SAHMRI's specific and opportunistic pathogen‐free animal care facility. Male mice were used exclusively to minimize the variability associated with fluctuations in gonadal hormones during the female oestrous cycle, which can modulate neuronal excitability and synaptic function (e.g., oestrous‐stage‐dependent differences in excitability in striatal neurons) (Krentzel et al., 2022). This approach also avoided baseline sex differences in neuronal properties (Iezzi et al., 2024) and hormonal influences on colonic sensitivity (Tramullas et al., 2021).

### 2.2 Human DRG

Four L1 DRGs were collected by AnaBios (San Diego, CA, USA) from human adult organ donors (22.2 ± 2.08 years of age; male:female ratio = 2:2). The samples were preserved in RNAlater (Thermo Fisher Scientific, Waltham, MA, USA) and shipped to SAHMRI for processing (Castro et al., 2017, 2019). The AnaBios NIH‐compliant ethics statement is available at: https://anabios.com/ethics‐statement.

### 2.3 Quantitative reverse‐transcription‐PCR

Total RNA was extracted from human L1 DRG or mouse thoracolumbar (T10–L1) DRGs using the PureLink RNA Isolation Kit (Thermo Fisher Scientific). Quantitative reverse‐transcription–PCR (qRT‐PCR) was conducted with 20 ng RNA/well using the EXPRESS One‐Step Superscript Kit (Thermo Fisher Scientific) and predesigned mouse‐ or human‐specific TaqMan probes for GABA_B_R subunits (*Gabbr1*, Mm00433461_m1 or Hs00559488_m1, *Gabbr2*, Mm01352554_m1 or Hs01554996_m1), Ca_V_2.2 (*Cacna1b*, Mm01333678_m1 or Hs01053090_m1), Ca_V_2.3 (*Cacna1e*, Mm00494444_m1 or Hs00167789_m1), GIRK1–4 (*Kcnj3*, Mm00434618_m1 or Hs04334861_s1, *Kcnj6*, Mm01215650_m1 or Hs01040524_m1, *Kcnj9*, Mm00434621_m1 or Hs05018005_m1, *Kcnj5*, Mm01175829_m1 or Hs00168476_m1), *Gapdh* (Mm99999915_g1), *Ppia* (Mm02342430_g1 or Hs99999904_m1), and *Actb* (Mm00607939_s1 or Hs99999903_m1). mRNA was transcribed into cDNA using the Superscript IV Kit (Thermo Fisher Scientific). All samples were measured in duplicate. Raw data were analysed using DA2 software (Thermo Fisher Scientific) and exported to Microsoft Excel to calculate relative mRNA quantities before transferring data into Prism (GraphPad Software, Boston, MA, USA) for graphical representation. The comparative cycle threshold method was used to quantify the abundance of target transcripts relative to the reference genes (*Gapdh, Ppia* and *Actb*).

### 2.4 Single‐cell RT‐PCR

A total of 53 single subserosal colon‐traced dissociated TL DRG neurons were isolated from one healthy mouse (C57Bl/6J; male; 16 weeks old) and collected using a micromanipulator at ×40 magnification. During the picking process, a continuous slow flow of sterile, RNA/DNAse‐free PBS was applied to minimize contamination with ambient RNA. After isolating a neuron, the glass capillary containing the cell was broken into a tube containing 10 µL lysis buffer with DNAse (TaqMan Gene Expression Cells‐to‐CT Kit; Thermo Fisher Scientific). The entire sample was used for cDNA synthesis (SuperScript VILO cDNA Synthesis Kit, Thermo Fisher Scientific) and diluted 1:3 to measure target expression per cell using RT‐PCR for 50 cycles. For each coverslip, a bath control was collected and analysed together with the samples. Expression of *Tubb3* (Mm00727586_s1) served as a positive control to confirm neuronal identity. Cells were excluded from analysis if they lacked *Tubb3* expression or tested positive for *Gfap* (Mm01253033_m1), indicating contamination with glial cells. Out of the initial 53 cells, 12 were excluded based on these criteria. The frequency of the expression of a particular target was calculated from the remaining 41 neurons.

### 2.5 Cell culture for patch clamp recordings of mouse DRGs

Mice were euthanised by inhalation of 5% isoflurane in a clear induction chamber until respiratory arrest, followed by decapitation as a secondary method, in accordance with institutional animal ethics guidelines. The thoracic and lumbar DRG were exposed via laminectomy, harvested and immediately transferred to ice‐cold (4°C) Ca^2+^‐ and Mg^2+^‐free Hanks’ buffered saline solution (HBSS). The DRGs were trimmed, removing central and peripheral nerve processes, and digested in HBSS containing collagenase type II (3 mg mL^−1^; Worthington Biomedical Corp., Lakewood, NJ, USA) and dispase (4 mg mL^−1^; Thermo Fisher Scientific) at 37°C for 40 min. Following digestion, the ganglia were rinsed 3 to 4 times with 37°C/5% CO_2_‐equilibrated F‐12/GlutaMAX (Thermo Fisher Scientific) supplemented with 10% heat‐inactivated FBS (Thermo Fisher Scientific) and 1% Pen/Strep. Cells were dispersed by mechanical trituration with progressively smaller fire‐polished glass Pasteur pipettes. The resulting cell suspension was filtered through a 160 µm nylon mesh (Millipore Australia Pty Ltd, North Ryde, NSW, Australia) to remove any undigested material. Dissociated DRG neurons were plated onto a 12 mm coverglass coated with poly‐d‐lysine (Sigma‐Aldrich, Melbourne, VIC, Australia) and allowed to settle for ∼3 h at 37°C, supplemented with fresh medium, and incubated overnight before patch‐clamp recordings.

### 2.6 Whole‐cell current‐clamp electrophysiology of mouse DRG neurons

Current‐clamp recordings of DRG neurons were carried out within 24 h of dissociation in an extracellular (bath) solution containing (in mM): 140 NaCl, 4 KCl, 2 CaCl_2_, 1 MgCl_2_, 10 HEPES, and 10 glucose; pH 7.4 adjusted with NaOH (∼320 mOsmol kg^−1^). Fire‐polished borosilicate patch pipettes (World Precision Instruments, Sarasota, FL, USA) had resistances of 2–4 MΩ when filled with an internal solution containing (in mM): 130 KCl, 20 NaCl, 5 EGTA, 5 MgCl_2_, 10 HEPES, 5 Mg‐ATP, and 0.2 Na_2_GTP; pH 7.2 adjusted with KOH (∼300 mOsmol kg^−1^). Membrane voltage and current were recorded using a MultiClamp 700B amplifier and digitized with a Digidata 1440A (Molecular Devices, San Jose, CA, USA). Whole‐cell configuration was achieved in voltage‐clamp mode before transitioning to current clamp mode. Depolarizing current steps of 500 ms duration at 0.2 Hz were applied to elicit action potential firing in small‐ to medium diameter (<30 µm) DRG neurons with resting membrane potentials (RMP) more negative than −40 mV.

Modulation of high voltage‐activated N‐type (Ca_V_2.2) channels by ω‐conotoxin CVIE and GIRK channels by the antagonist tertiapin‐Q (TPQ) and the agonist ML297 was conducted via bath application of these compounds. In a series of experiments, the GABA_B_R agonist, α‐conotoxin Vc1.1, was co‐applied with either CVIE or ML297. All drugs were dissolved in distilled H_2_O to prepare stock solutions at the appropriate concentration. The extracellular solutions were superfused using a peristaltic pump at a flow rate of 1 mL min^−1^ in a ∼500 µL experimental chamber maintained at room temperature (21–23°C). TPQ was purchased from Abcam (Cambridge, UK), ML297 was obtained from Tocris (Bristol, UK), and ω‐conotoxin CVIE and α‐conotoxin Vc1.1 were synthesized as described previously (Berecki et al., 2010) and kindly provided by Professors David Craik and Richard Clark (The University of Queensland, Brisbane, Australia).

### 2.7 Mouse model of chronic visceral hypersensitivity

Colitis was induced by administering 2,4‐dinitrobenzene sulfonic acid (DNBS) as described previously (Castro et al., 2022). Briefly, 13‐ to 15‐week‐old mice were anaesthetised with isoflurane (3%–4% for induction and 1%–2% for maintenance) and received a single intra‐colonic enema of 100 µL DNBS/ethanol solution (5 mg DNBS dissolved in 100 µL of 30% ethanol) via a polyethylene catheter to induce colonic inflammation (Castro et al., 2013, 2019). Histological examination of mucosal architecture, cellular infiltrate, crypt abscesses, and goblet cell depletion confirmed significant DNBS‐induced damage by day 3 post‐treatment. This damage largely resolved spontaneously by day 7 and was fully resolved by day 28 (Hughes et al., 2009). High‐threshold colonic nociceptors from mice at the 28‐day time point displayed significant mechanical hypersensitivity and reduced mechanical activation thresholds (Castro et al., 2018, 2019; de Araujo et al., 2014; Hughes et al., 2009; Osteen et al., 2016; Salvatierra et al., 2018). This model is characterized by hyperalgesia and allodynia in response to colorectal distension and is therefore termed ‘CVH’ (Castro et al., 2022, 2019; Kremsmayr et al., 2024; Osteen et al., 2016; Salvatierra et al., 2018). CVH mice were euthanised 28 days after DNBS treatment by CO_2_ inhalation for 8 min in a QuietTek Euthanasia Station (Next Advance Inc., Troy, NY, USA), which constituted the sole method of termination. No anaesthetic agent was administered prior to CO_2_ exposure.

### 2.8 Retrograde tracing to identify colon‐innervating DRG neurons

Cholera Toxin subunit B conjugated to AlexaFluor‐488 or ‐555 (Thermo Fisher Scientific) was injected (4 µL/injection) subserosally at three sites within the distal colon of healthy mice (for single‐cell RT‐PCR and patch‐clamp recordings) or CVH mice (patch‐clamp recordings) using a Hamilton syringe fitted with a 23‐gauge needle, as previously described (Harrington et al., 2019).

### 2.9 Cell culture for patch clamp recordings and single‐cell picking of colonic DRGs

Four days after retrograde tracing, mice were euthanized by CO_2_ inhalation, and thoracolumbar (TL, from T9–L1) DRGs were removed and dissociated (Castro et al., 2017, 2019). DRGs were enzymatically digested first with 4 mg mL^−1^ collagenase II (Thermo Fisher Scientific) and 5.3 mg mL^−1^ dispase I (Thermo Fisher Scientific) for 30 min at 37°C, followed by a second digestion with 4 mg mL^−1^ collagenase II for 10 min at 37°C. The dissociated neurons were resuspended in DMEM (Thermo Fisher Scientific) supplemented with 10% fetal calf serum (Thermo Fisher Scientific), 2 mM l‐glutamine (Thermo Fisher Scientific), 100 µM MEM non‐essential amino acids (Thermo Fisher Scientific), 96 ng mL^−1^ nerve growth factor, and 100 mg mL^−1^ penicillin/streptomycin (Thermo Fisher Scientific). Neurons were spot‐plated onto coverslips coated with laminin (20 µg mL^−1^; Sigma‐Aldrich) and poly‐d‐lysine (800 µg mL^−1^; Thermo Fisher Scientific) and maintained in an incubator at 37°C with 5% CO_2_.

### 2.10 Whole‐cell current‐clamp electrophysiology of colon‐innervating DRG neurons

Dissociated DRG neurons isolated from colon‐traced healthy or CVH mice were recorded on day 1 post‐culture (20–30 h after plating) (Castro et al., 2018; Grundy et al., 2018; Salvatierra et al., 2018). The intracellular current‐clamp solution contained (in mM): 135 KCl, 2 MgCl_2_, 2 MgATP, 5 EGTA‐Na, and 10 HEPES‐Na; adjusted to pH 7.3. The extracellular (bath) current‐clamp solution contained (in mM): 140 NaCl, 4 KCl, 2 MgCl_2_, 2 CaCl_2_, 10 HEPES, and 5 glucose; adjusted to pH 7.4. Standard‐wall borosilicate glass pipettes (OD × ID × length: 1.5 mm × 0.86 mm × 7.5 cm; Harvard Apparatus, Holliston, MA, USA) pulled and fire‐polished to a resistance of 3–6 MΩ using a P‐97 (Sutter Instrument Co., Novato, CA, USA) pipette puller was used. The protocol used was as follows: in current‐clamp mode, neurons were held at −70 mV for 15 ms, hyperpolarized with a −20 pA current injection for 475 ms, then returned to –70 mV for 100 ms. Stepwise depolarizing pulses were applied in 0 pA or 25 mV increments (475 ms) from a holding potential of –70 mV, with 2 s repetition intervals, to determine the rheobase (minimum amount of current required to elicit an action potential). Rheobase was assessed in both the presence and the absence of TPQ (100 nM) or the HCN inhibitor ZD7288 (50 µM). Neurons with a resting membrane potential more depolarized than –40 mV were excluded from recordings, as this indicated poor cell health. Recordings were amplified using an Axopatch 200A, digitized with a Digidata 1322A, sampled at 20 kHz, filtered at 5 kHz, and recorded with pCLAMP 9 software (Molecular Devices). Data were analysed using Clampfit 10.3.2 (Molecular Devices) and Prism v9.3.1.

### 2.11 *Ex vivo* electrophysiology

Recordings were obtained from healthy male C57BL/6J mice. Pelvic and splanchnic afferent nerve activity was measured *ex vivo* using an isolated segment of the mouse colon ligated at either end to allow for fluid distension (Bayrer et al., 2023). The tissue was mounted in a custom‐built organ bath and perfused with warm, carbogenated physiological Krebs buffer (composition in mmol/L: 118.4 NaCl, 24.9 NaHCO_3_, 1.9 CaCl_2_, 1.2 MgSO_4_, 4.7 KCl, 1.2 KH_2_PO_4_, 11.7 glucose). To suppress smooth muscle activity, the Krebs solution included the L‐type calcium channel antagonist nifedipine (1 µM). The prostaglandin synthesis inhibitor indomethacin (3 µM) was also added to suppress potential inhibitory actions of endogenous prostaglandins. The pelvic and splanchnic nerves were carefully isolated and placed within a sealed glass pipette containing a microelectrode (WPI) connected to a NeuroLog headstage (NL100AK; Digitimer Ltd, Welwyn Garden City, UK). Nerve activity was amplified (NL104), filtered (NL 125/126, bandpass 50–5000 Hz, NeuroLog; Digitimer), and digitised using a CED 1401 interface (Cambridge Electronic Design, Cambridge, UK) for offline analysis with Spike2 software (Cambridge Electronic Design). Nerve activity was measured at baseline and during ramp distension with Krebs solution or TPQ (flow rate: 100 µL min^−1^; pressure range: 0 to 80 mmHg), measured with a pressure transducer (NL108T2, Digitimer).

The number of action potentials exceeding a preset threshold (twice the background electrical noise) was determined per second to quantify the afferent response. Responses were compared before and after the intraluminal application of TPQ (10 and 100 µM). Single‐unit analysis of action potentials was conducted offline by matching individual spike waveforms through linear interpolation using Spike2 v10.05 software (Cambridge Electronic Design).

### 2.12 Statistical analysis

Data are expressed as means ± SD or as the percentage of neurons. Figures were prepared using GraphPad Prism 9 software. *N* represents the number of animals, and *n* denotes the number of neurons or afferents. Statistical significance was defined as *P* < 0.05 (*), *P* < 0.01 (**), *P* < 0.001 (***) and *P* < 0.0001 (****). All data were analysed using GraphPad Prism 9 and assessed for normal distribution using either the Kolmogorov–Smirnov or Shapiro–Wilk test. Depending on the experimental design, data were further analysed using one‐way ANOVA followed by Tukey's *post hoc* test for multiple group comparisons, two‐way ANOVA followed by Bonferroni's *post hoc* test, or Student's paired or unpaired two‐tailed *t*‐test. The specific statistical tests used for each dataset are detailed in the corresponding figure legends.

## 3 RESULTS

### 3.1 Expression of GABA_B_R subunits, Ca_V_2.2, Ca_V_2.3 and GIRK1–4 in mouse and human thoracolumbar DRGs

**FIGURE 1:**
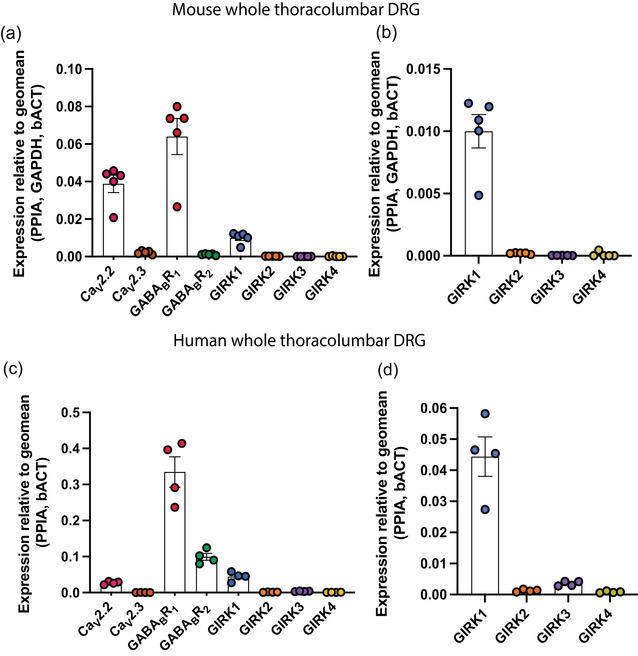
Expression patterns of GABA_B_ receptors and associated ion channels in mouse and human thoracolumbar dorsal root ganglion (DRG) (T10−L1). (a) Quantitative RT‐PCR (QRT‐PCR) analysis showing high mRNA abundance of GABA_B_R1, Ca_V_2.2, and GIRK1 in whole mouse thoracolumbar DRG. Data represent five individual mice (*N* = 5), with each dot representing one mouse. (b) Items from (a) shown on an expanded scale, showing QRT‐PCR analysis of GIRK1−4 mRNA expression in whole mouse thoracolumbar DRG (T10−L1). Results indicate low mRNA abundance for GIRK2−4. (c) QRT‐PCR analysis of mRNA expression in whole human thoracolumbar (L1). Results show high expression of GABA_B_R1, GABA_B_R2 and GIRK1, while Ca_V_2.2 is expressed at relatively lower levels compared to the abundant GABA_B_R1 expression. (d) Items from (c) shown on an expanded scale, showing QRT‐PCR analysis of GIRK1−4 expression in human L1 DRG, indicating low mRNA abundance of GIRK2−4. Data are from DRG samples of four human donors, with each point representing an individual donor (*N* = 4). bACT, β‐actin; GAPDH, glyceraldehyde 3‐phosphate dehydrogenase; PPIA, peptidylprolyl isomerase A.

Quantitative RT‐PCR (QRT‐PCR) analysis of whole mouse DRG samples revealed that GABA_B_R1, Ca_V_2.2 and GIRK1 were the most abundant targets expressed in the thoracolumbar dorsal root ganglia (DRGs, T10–L1, *N* = 5 mice), followed by Ca_V_2.3 and GABA_B_R2. In contrast, GIRK2–4 exhibited very low mRNA expression levels, 51 times lower than GIRK1 for GIRK2, 106 times lower for GIRK4, and 306 times lower for GIRK3 (Figure 1a, b). In mouse DRGs, the fold change in expression for each target relative to the least expressed gene was as follows: GABA_B_R1 (1961) > Ca_V_2.2 (1188) > GIRK1 (306) > Cav2.3 (66) > GABA_B_R2 (34) > GIRK2 (6) > GIRK4 (3) > GIRK3 (1). The expression pattern observed in human thoracolumbar DRGs was somewhat different, with GABA_B_R2 expression relatively higher in human DRGs (*N* = 4 donors), being four‐fold lower than GABA_B_R1, compared to mouse DRGs, where GABA_B_R2 was 60‐fold lower than GABA_B_R1 (Figure 1c, d). Additionally, the expression of Cav2.3 in human DRGs was notably lower than in mice, with Ca_V_2.3 being the least expressed target in human DRGs. Similar to mouse TL DRG, Cav2.2 was highly expressed in human L1 DRG; however, the ratio of GABA_B_R1 to Ca_V_2.2 was only 12 to 1 compared to the similar expression levels in mice (1.6:1). In human DRGs, the fold change in expression for each target relative to the least expressed gene was as follows: GABA_B_R1 (1231) > GABA_B_R2 (365) > GIRK1 (163) > Ca_V_2.2 (99) > GIRK3 (13) > GIRK2 (5) > GIRK4 (3) > Ca_V_2.3 (1). Overall, these findings highlight that Ca_V_2.2, GABA_B_R1, and GIRK1 are the dominant isoforms in both mouse and human DRGs, suggesting their potential functional importance in thoracolumbar sensory processing.

### 3.2 Co‐expression of GABA_B_R subunits, Ca_V_2.2, Ca_V_2.3 and GIRK channels in colon‐innervating DRGs

**FIGURE 2:**
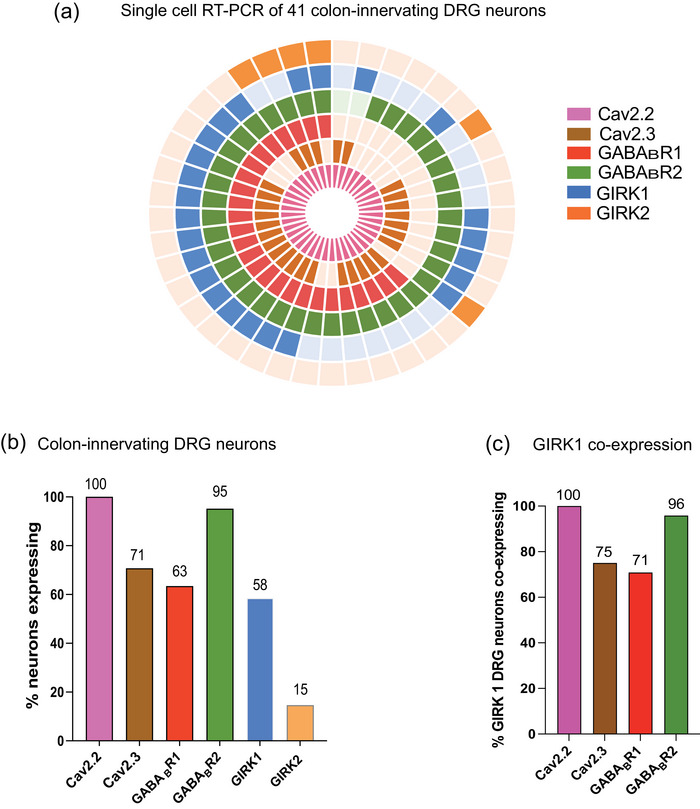
Expression and co‐expression of ion channel and receptor genes in retrogradely traced colon‐innervating mouse dorsal root ganglion (DRG) neurons. (a) Doughnut plot illustrating the expression and co‐expression of genes encoding Ca_V_2.2, Ca_V_2.3, GABA_B_R1, GABA_B_R2 and GIRK1−2 in 41 individual retrogradely traced colon‐innervating DRG neurons (*N* = 1 mouse). Each gene is represented by a distinct colour, with expression indicated by bold shading. GIRK2 is depicted in the outer ring, while the inner ring represents Ca_V_2.2. Individual neurons are arranged radially, allowing for clear visualisation of gene co‐expression within a single neuron from the outermost to the innermost rings. Some neurons expressed most targets, while others expressed only specific combinations. GIRK3 or GIRK4 were not detected and are therefore not shown. (b) Single‐cell RT‐PCR analysis of retrogradely traced colon‐innervating DRG neurons, showing the percentage of colon‐innervating DRG neurons expressing each transcript: Ca_V_2.2 (100%), Ca_V_2.3 (71%), GABA_B_R1 (63%), GABA_B_R2 (95%), GIRK1 (58%), and GIRK2 (15%). No expression of GIRK3 or GIRK4 was detected. (c) Single‐cell RT‐PCR analysis highlighting the gene co‐expression of GIRK1 with Ca_V_2.2 (100%), Ca_V_2.3 (75%), GABA_B_R1 (71%), and GABA_B_R2 (96%).

Single‐cell RT‐PCR from retrogradely traced mouse colon‐innervating DRGs was performed to assess the expression of GABA_B_R, Ca_V_ channels, and GIRK channels specifically in colonic DRG neurons. The analysis revealed high expression levels of Ca_V_2.2 and GABA_B_R2, detected in 100% and 95% of colon‐innervating DRG neurons, respectively (*n* = 41) (Figure 2a, b). In contrast, Ca_V_2.3 was expressed in 29 of 41 cells (71%), and GABA_B_R1 was expressed in 26 of 41 neurons (63%, Figure 2a, b). Among the 41 colonic neurons analysed, GIRK1 was expressed in 24 neurons (58%), whereas GIRK2 was detected in only six neurons (15%). GIRK3 and GIRK4 expression levels were below the detection threshold and were not observed in any of the neurons analysed. Co‐expression analysis revealed that when GIRK1 was expressed it was highly co‐expressed with Ca_V_2.2 (100%), Ca_V_2.3 (75%), GABA_B_R1 (71%), and GABA_B_R2 (96%) (Figure 2c), suggesting a strong functional link between GABA_B_R signalling, Ca_V_ channels, and GIRK channel activity in colonic sensory DRG neurons.

### 3.3 Effects of the selective Ca_V_2.2 antagonist ω‐conotoxin CVIE and the GIRK channel agonist ML297 on mouse DRG neuronal excitability

**FIGURE 3:**
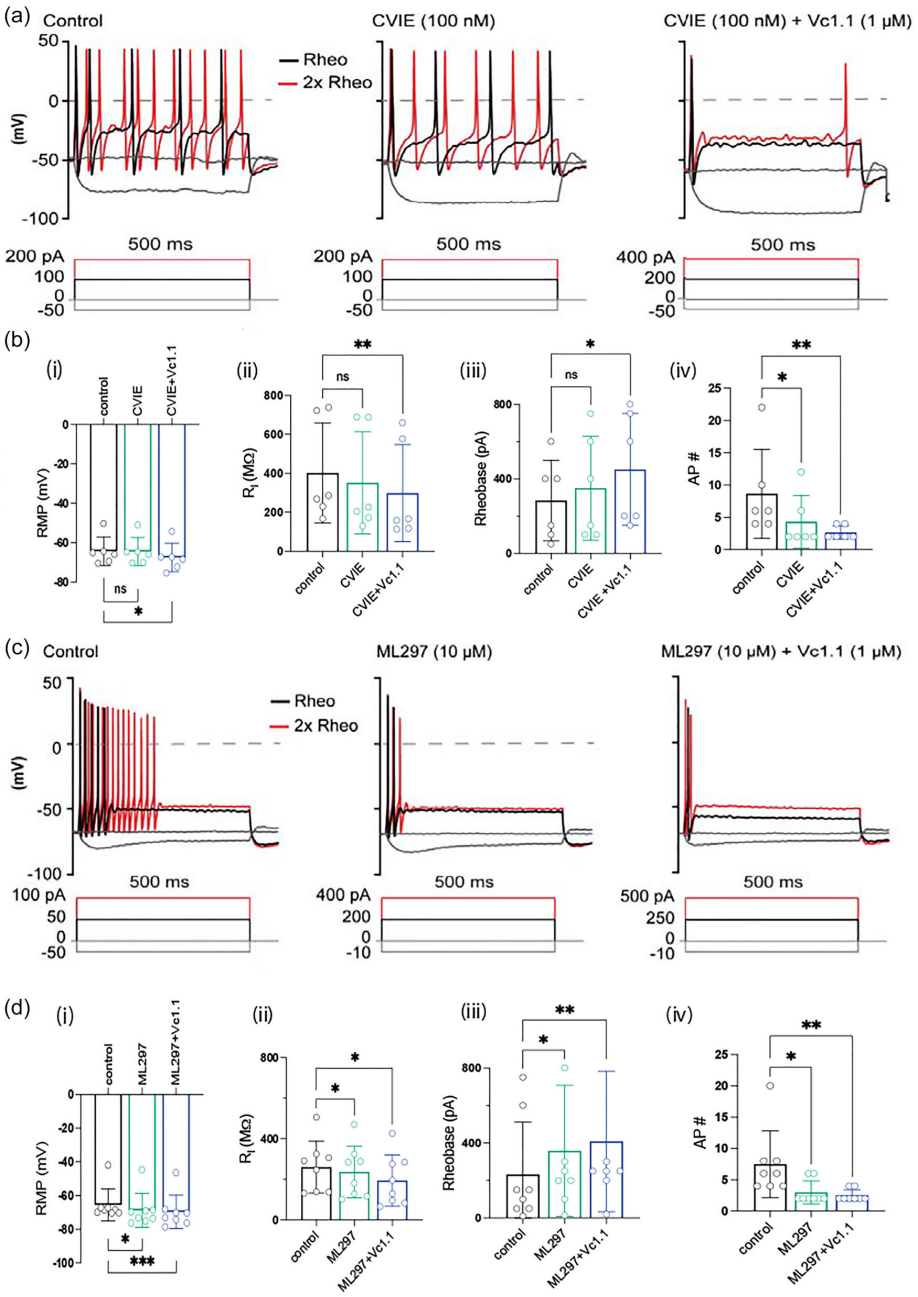
Effects of ω‐conotoxin CVIE, ML297 and α‐conotoxin Vc1.1 on the electrophysiological properties of adult mouse dorsal root ganglion (DRG) neurons. (a) Representative voltage responses to current clamp steps recorded in a small diameter DRG neuron (23 µm) in the absence (control; left) and presence of CVIE (100 nM) (middle) and CVIE (100 nM) + Vc1.1 (1 µM) (right). The dashed line indicates 0 mV. Rheobase (black) and 2× rheobase (red) are shown for both membrane potential and current. (b) Bar graphs summarizing the effects of CVIE and CVIE + Vc1.1 on (i) resting membrane potential (RMP, ns *P* = 0.999, *P = 0.012), (ii) input resistance (*R*_i_, ns *P* = 0.496, **P = 0.003), (iii) rheobase (ns *P* = 0.456, *P = 0.022), and (iv) AP frequency (Hz) (*P = 0.042, **P = 0.008) in response to 500 ms depolarizing current steps in small to medium diameter DRG neurons from adult mice. (c) Representative voltage responses to current clamp steps recorded in a mouse DRG neuron (24.5 µm) in the absence (control; left) and presence of ML297 (10 µM) (middle), and ML297 (10 µM) + Vc1.1 (1 µM) (right). The dashed line denotes 0 mV. Rheobase (black) and 2× rheobase (red) are shown for both membrane potential and current. (d) Bar graphs summarizing the effects ML297 and ML297 + Vc1.1 on (i) resting membrane potential (RMP, *P = 0.025, ***P = 0.0009), (ii) input resistance (*R*_i_, *P = 0.017, *P = 0.013), (iii) rheobase (*P = 0.017, **P = 0.001), and (iv) AP frequency (Hz, *P = 0.012, **P = 0.002) in response to a 500 ms depolarizing current step at 2× rheobase in adult mouse DRG neurons (<30 µm diameter). Data are presented as means ± SD, with the number of neurons indicated within each bar (*n* = 6 (b), *n* = 8 (d)). Statistical significance was determined using one‐way repeated measures ANOVA or Friedman's test. ns, not significant, *P < 0.05, **P < 0.001, ***P < 0.0001.

To investigate the roles of Ca_V_2.2 and GIRK channels in regulating neuronal excitability, whole‐cell patch‐clamp recordings were performed on mouse DRG neurons in the presence of the selective Ca_V_2.2 antagonist ω‐conotoxin CVIE and the GIRK channel agonist ML297 (Figure 3). Application of ω‐conotoxin CVIE (100 nM) significantly reduced action potential (AP) firing frequency by approximately 50% (*P* = 0.042) and increased rheobase by approximately 20% (*P* = 0.456), without affecting the resting membrane potential (−64.5 ± 2.9 mV, *n* = 6; *P* = 0.999) (Figure 3a, b). Co‐application of ω‐conotoxin CVIE with α‐conotoxin Vc1.1 caused membrane hyperpolarization and a further reduction in AP firing (Figure 3a). Together, co‐application of ω‐conotoxin CVIE and α‐conotoxin Vc1.1 significantly decreased the resting membrane potential (*P* = 0.012) and input resistance (*P* = 0.003), reduced AP firing frequency (*P* = 0.008), and significantly increased rheobase (*P* = 0.022) (*n* = 8) (Figure 3b), indicating a synergistic suppression of excitability in DRG neurons.

Similarly, application of the GIRK channel agonist ML297 (10 µM) produced effects comparable to ω‐conotoxin CVIE on resting membrane potential (*P* = 0.025), rheobase (*P* = 0.017), input resistance (*P* = 0.017), and AP firing frequency (*P* = 0.012) (Figure 3c). However, unlike ω‐CVIE, ML297 caused significant membrane hyperpolarization (>3.5 mV, *n* = 8; *P* = 0.025). These findings are consistent with previous studies showing that GABA_B_R‐dependent GIRK channel potentiation by baclofen or Vc1.1 causes membrane hyperpolarization and reduced excitability in mouse DRG neurons (Bony et al., 2022). Furthermore, co‐application of ML297 and Vc1.1 (1 µM) produced an additive effect, further increasing the current required to induce AP firing (*P* = 0.001) and significantly reducing firing frequency in healthy mouse DRG neurons (*P* = 0.002) (*n* = 8) (Figure 3c, d; Table 1).

**TABLE 1:** Passive and active electrical properties of adult mouse DRG neurons in the absence (control) and presence of either ω‐conotoxin CVIE (100 nM) or ML297 (10 µM) applied alone or in combination with α‐conotoxin Vc1.1 (1 µM).

	Control	ω‐CVIE	ω‐CVIE + Vc1.1	*n*	Control	ML297	ML297 + Vc1.1	*n*
**Rheobase (pA)**	283.3 ± 88.2	350 ± 14.0	450 ± 122.5	6	232.5 ± 99.1	358.1 ± 123.7	408.8 ± 132.4	8
**Input resistance (MΩ)**	401.7 ± 105.0	351.4 ± 107.3	298.4 ± 101.9	6	260.9 ± 42.1	237.3 ± 45.2	195.0 ± 44.5	8
**RMP (mV)**	−64.3 ± 2.9	−64.5 ± 2.9	−43.4 ± 23.3	6	−65.6 ± 3.4	−68.8 ± 3.6	−69.7 ± 3.5	8
**AP (Hz)**	8.7 ± 2.8	4.3 ± 1.7	2.7 ± 0.4	6	7.5 ± 1.9	3.0 ± 0.7	2.5 ± 0.3	8

Parameters analysed include resting membrane potential (RMP, mV), input resistance (*R*_i_, MΩ), rheobase (pA), and action potential (AP) frequency (Hz) in response to a 500 ms depolarizing current step. Results are presented as means ± SD, with the number of experiments indicated. Statistical significance was determined using one‐way repeated measures ANOVA or Friedman's test.

### 3.4 GIRK channel inhibitor TPQ increases the excitability of adult mouse DRG neurons, and its effects are counteracted by α‐conotoxin Vc1.1

**FIGURE 4:**
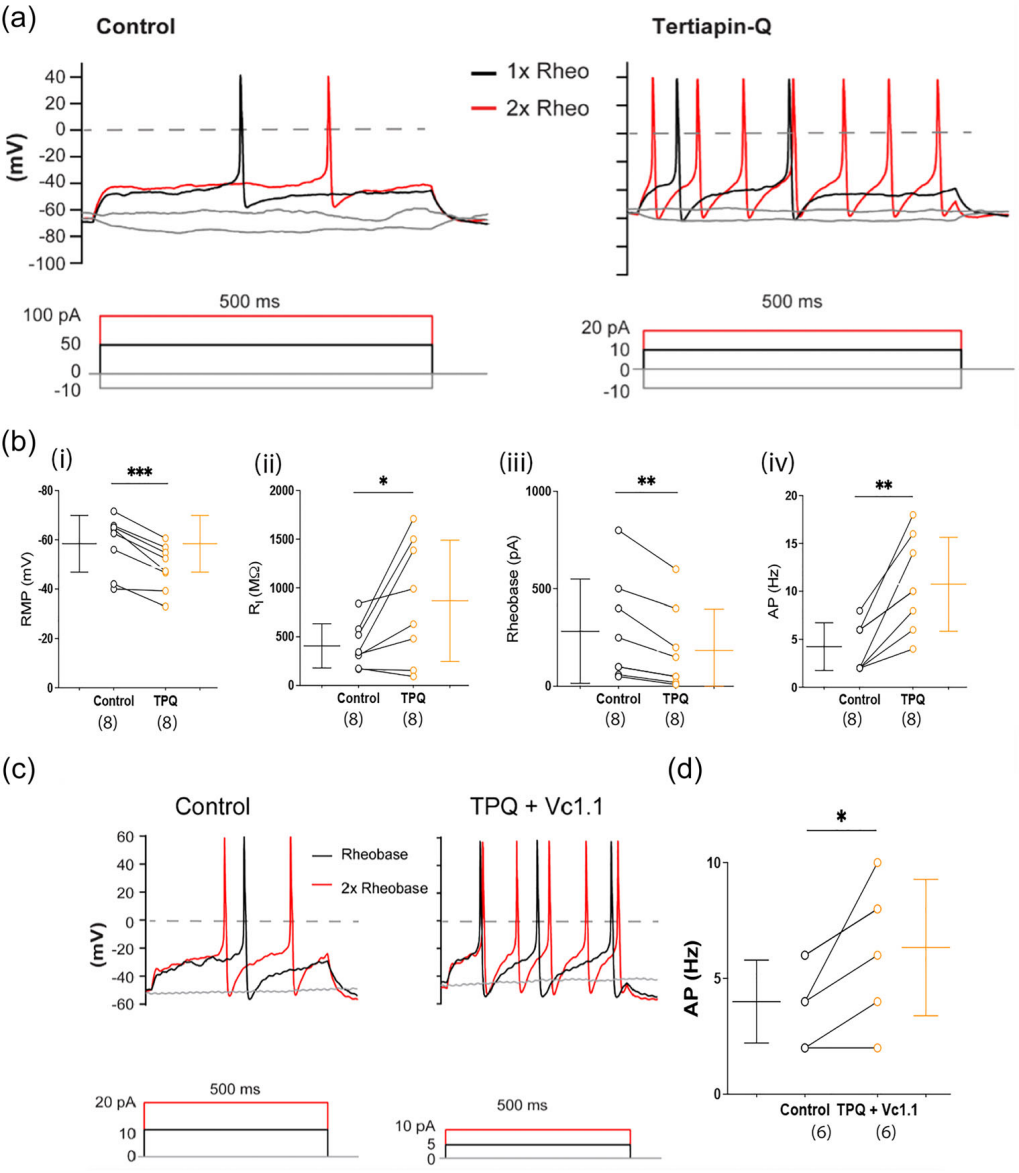
The GIRK channel inhibitor tertiapin‐Q (TPQ) enhances the excitability of adult mouse dorsal root ganglion (DRG) neurons. (a) Representative voltage responses to current clamp steps recorded in a mouse DRG neuron (26 µm diameter) in the absence (control; left) and presence of 100 nM TPQ (right). The dashed line denotes 0 mV. Rheobase (black) and 2× rheobase (red) are shown for both membrane potential and injected current. (b) Paired scatter dot plots illustrating the effects TPQ (100 nM) on (i) resting membrane potential (RMP, ***P = 0048), (ii) input resistance (*R*_i_, *P = 0.036), (iii) rheobase (nA, **P = 0.0048), and (iv) AP frequency (Hz, **P = 0.008) in response to a 500 ms depolarizing current step at 2× rheobase in small‐ to medium‐diameter (<30 µm) adult mouse DRG neurons. (c, d) Lack of effect of α‐conotoxin Vc1.1 on neuronal excitability in the presence of TPQ. Representative voltage responses to current clamp steps recorded in a small‐ to medium‐diameter mouse DRG neuron recorded in the absence (control) and presence of 100 nM TPQ + 1 µM α‐conotoxin Vc1.1 (c). Insets show responses to 500 ms depolarizing current steps. The dashed line denotes 0 mV, and rheobase (black) and 2× rheobase (red) are indicated for both membrane potential and current. (d) Paired scatter dot plot showing action potential (AP) frequency (Hz) at 2× rheobase under control conditions and in the presence of TPQ (100 nM) + Vc1.1 (1 µM) in small‐ to medium‐diameter (<30 µm) adult mouse DRG neurons (*P = 0.33). Data are presented as the mean ± SD, with the number of neurons indicated in parentheses (*n* = 8 (b), *n* = 6 (d)). Statistical analysis was performed using Student's paired *t*‐test or Wilcoxon's matched‐pairs signed rank test; *P < 0.05, **P < 0.001, ***P < 0.0001.

To further examine the role of GIRK channels in regulating neuronal excitability, we applied the specific GIRK channel antagonist TPQ (100 nM) to adult mouse DRG neurons. TPQ application caused membrane depolarization and a significant increase in neuronal excitability (Figure 4a, b; Table 1). Specifically, TPQ significantly elevated the resting membrane potential (*P* = 0.0005) and input resistance (*P* = 0.036), increased action potential firing frequency (*P* = 0.008) and reduced rheobase (*P* = 0.0048) (*n* = 8). To determine whether TPQ could interfere with GIRK channel potentiation by α‐conotoxin Vc1.1, TPQ and Vc1.1 were co‐applied. Co‐incubation increased AP discharge relative to Vc1.1 alone, indicating that TPQ blocked the potentiation of GIRK channel currents induced by Vc1.1 (*P* = 0.033, *n* = 6). Under control conditions, Vc1.1 potentiates GIRK channel activity, leading to reduced AP firing. Thus, the excitatory effects observed with TPQ in the presence of Vc1.1 suggest that GIRK channel inhibition reverses the Vc1.1‐induced suppression of neuronal excitability (Figure 4c, d).

### 3.5 GIRK channel inhibitor TPQ does not affect the responsiveness of mouse colonic afferents

**FIGURE 5:**
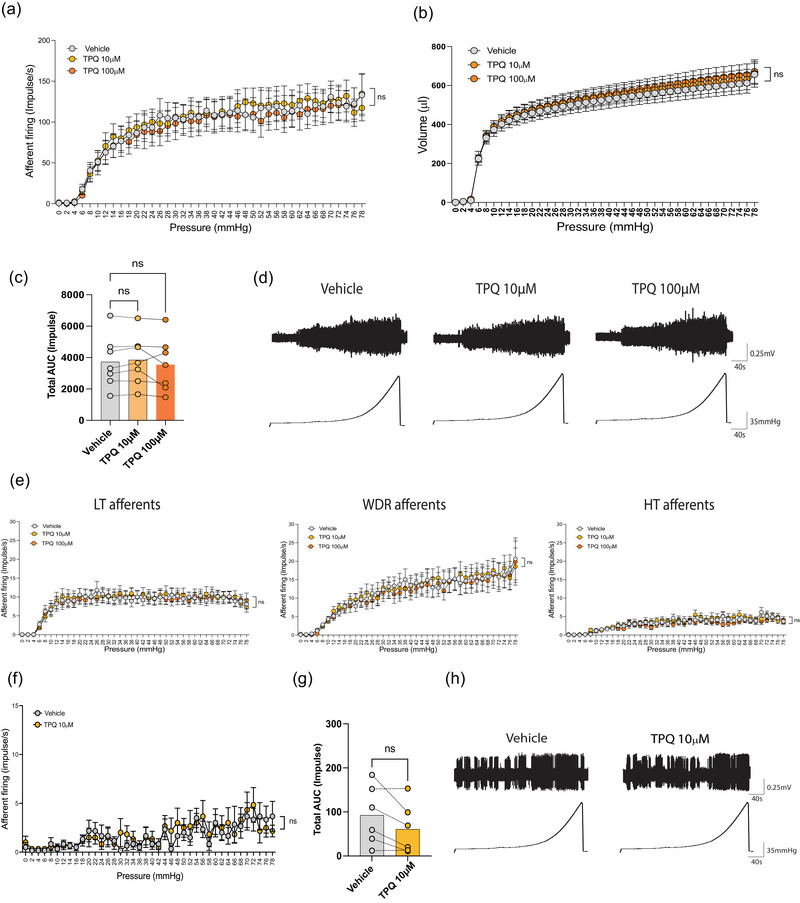
Lack of effect of tertiapin‐Q (TPQ) on pelvic and splanchnic afferent responses to colonic distension. (a, b) Group data showing pelvic nerve afferent firing responses (a) and colonic compliance (b) to increasing distension pressures in *ex vivo* preparations from healthy C57BL/6J mice (*N* = 7). Responses were recorded at baseline (vehicle‐treated Krebs distension) and following intraluminal application of TPQ at 10 or 100 µM. (c) Comparison of the total area under the curve (AUC) for afferent firing between vehicle and TPQ intraluminal application, showing no significant differences (ns, *P* = 0.208 for vehicle vs. TPQ 10 µM; *P* = 0.689 for vehicle vs. TPQ 100 µM). (d) Representative traces of pelvic nerve afferent firing in response to graded colonic distension in the absence (vehicle, left) and following (right) TPQ application (10 and 100 µM). (e) *Post hoc* single‐unit analysis of multiunit *ex vivo* colonic recordings. Pelvic nerve afferents were classified based on their discharge profile during ramp distension into low‐threshold (LT), wide dynamic range (WDR) or high‐threshold (HT). Total AUC for LT (*n* = 33), WDR (*n* = 25) and HT (*n* = 24) afferents during graded distension of the colon showed no significant effect of TPQ (10 or 100 µM). (f) Group data showing splanchnic nerve afferent firing responses to increasing colonic distension pressures in *ex vivo* preparations from healthy C57BL/6J mice (*N* = 6). Responses were recorded under baseline conditions (vehicle‐treated Krebs distension) and following intraluminal application of TPQ (10 µM). (g) Comparison of total AUC for splanchnic afferent firing between vehicle‐treated and TPQ‐treated conditions, showing no significant differences (ns, *P* = 0.059). (h) Representative traces of splanchnic nerve afferent firing in response to colonic distension in the absence (vehicle, left) and presence of TPQ (10 µM, right). Data are presented as before and after values. *P*‐values were determined using two‐way ANOVA (Šidák's multiple‐comparisons test) or one‐way ANOVA (Friedman's multiple‐comparisons test) as appropriate. ns, not significant.

To evaluate the functional role of GIRK channels in colonic sensory pathways, we conducted *ex vivo* recordings of pelvic and splanchnic nerve activity in response to colonic distension. Afferent recordings from pelvic nerves revealed that as intraluminal pressure increased, action potential firing also increased proportionately (Figure 5a). However, comparison of responses during colonic distension in the presence of vehicle (Krebs solution) versus intraluminal infusion of TPQ (10 or 100 µM) showed no significant differences in afferent firing rates (*P* = 0.208 for vehicle vs. TPQ 10 µM: *P* = 0.689 for vehicle vs. TPQ 100 µM) or colonic compliance (*P* = 0.249 for vehicle vs. TPQ 10 µM: *P* = 0.325 for vehicle vs. TPQ 100 µM) (*N* = 7) (Figure 5a, d). These findings indicate that GIRK channel inhibition by TPQ does not affect the excitability of healthy colonic pelvic afferents responding to mechanical distension. A more detailed analysis of individual afferent responses was performed by classifying pelvic colonic afferents based on their firing characteristics at specific distension pressures (Bayrer et al., 2023). TPQ treatment did not alter the mechanical sensitivity of low‐threshold (LT; *P* = 0.237 for vehicle vs. TPQ 10 µM; *P* = 0.192 for vehicle vs. TPQ 100 µM, *n* = 33), wide dynamic range (WDR; *P* = 0.469 for vehicle vs. TPQ 10 µM; *P* = 0.084 for vehicle vs. TPQ 100 µM, *n* = 25), or high‐threshold (HT; *P* = 0.513 for vehicle vs. TPQ 10 µM: *P* = 0.105 for vehicle vs. TPQ 100 µM, *n* = 24) pelvic afferents (Figure 5e), consistent with the whole pelvic nerve recordings.

We also examined the effects of TPQ on splanchnic afferent responses to colonic distension (Figure 5f, h). A small, non‐significant decrease in splanchnic afferent firing was observed following intracolonic infusion of TPQ (10 µM) compared to vehicle (Krebs solution, Figure 5f, g). Given that the majority of splanchnic afferents are high threshold, no further analysis of afferent subtypes was performed. Taken together, these results indicate that inhibiting GIRK channel activity with TPQ does not significantly affect the excitability of either pelvic or splanchnic colonic afferents in healthy mice during mechanical distension.

### 3.6 GIRK channel inhibition with TPQ does not affect the excitability of colon‐innervating DRG neurons in CVH states

**FIGURE 6:**
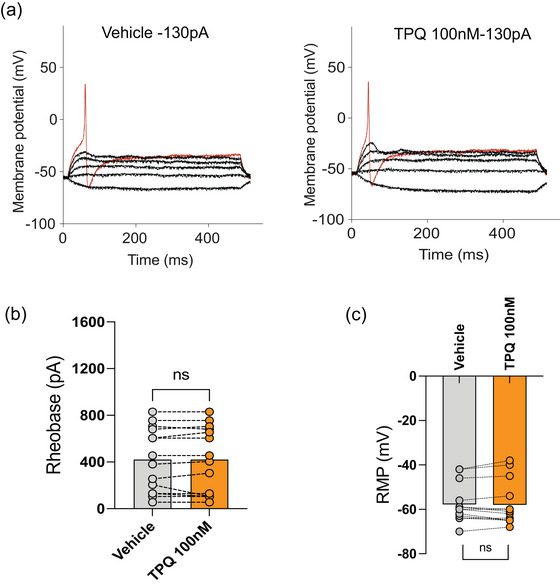
Lack of effect of tertiapin‐Q (TPQ) on the excitability of mouse colon‐innervating dorsal root ganglion (DRG) neurons in CVH states. (a) Representative voltage responses to depolarizing current clamp steps recorded from a thoracolumbar (TL) DRG neuron in the absence (control) and presence of TPQ (100 nM). Rheobase is indicated in red (130 pA). (b, c) Bar graphs showing no significant effect of TPQ (100 nM) on rheobase (ns, *P* = 0.999) or resting membrane potential (RMP, ns, *P* = 0.677) in colon‐innervating DRG neurons. Data are presented as before and after values for each neuron. *P*‐values were determined using a paired *t*‐test. *n* = 14 neurons from *N* = 3 mice.

To further assess the role of GIRK channels in regulating the excitability of colonic DRG neurons, whole‐cell patch‐clamp recordings were performed in the CVH model. Recordings were obtained from retrogradely traced colonic DRG neurons isolated from CVH mice in the presence of the GIRK channel inhibitor TPQ (100 nM). In contrast to our observations in general DRG neurons, TPQ application did not significantly alter the amount of injected current required to evoke an action potential, indicating no change in rheobase (*P* = 0.999, *n* = 14, *N* = 3) (Figure 6a, b). Furthermore, TPQ had no significant effect on the resting membrane potential of colonic DRG neurons from CVH mice (*P* = 0.677, *n* = 14, *N* = 3) (Figure 6c). These findings suggest that GIRK channel inhibition does not influence action potential firing in colon‐innervating DRGs under CVH conditions. Taken together with our findings in healthy tissue, this suggests that GIRK channels do not play a prominent role in modulating colonic neuronal excitability in either physiological or pathophysiological states.

### 3.7 The specific HCN channel inhibitor ZD7288 increases the excitability of healthy colonic DRG neurons

**FIGURE 7:**
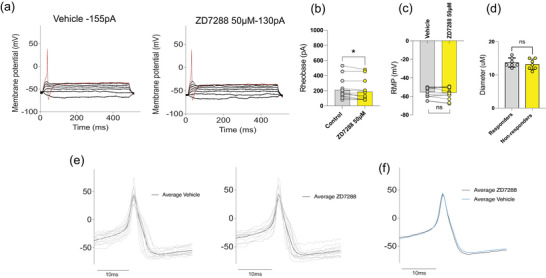
The HCN channel blocker ZD7288 increases the excitability of mouse colon‐innervating dorsal root ganglion (DRG) neurons. (a) Representative voltage responses to current clamp steps recorded from a colon‐innervating DRG neuron in the absence (control, vehicle) and presence of ZD7288 (50 µM). Rheobase is indicated in red. (b, c) Bar graphs showing the effects of ZD7288 on rheobase (*P = 0.014) and resting membrane potential (RMP; ns, *P* = 0.398) in colon‐innervating DRG neurons. *P‐*values were determined using a paired *t*‐test. Data are presented as before and after values for each neuron. (d) Bar graphs showing the soma diameter of neurons of responders and non‐responders to ZD7288 (ns, *P* = 0.508). Data are presented as means ± SD. (e) Representative individual action potential (AP) traces recorded under each condition (grey), with the average waveform overlaid in black and aligned to the peak depolarisation. (f) Overlay of average AP waveforms showing no significant differences between vehicle (blue) and ZD7288 (black). *n* = 14 neurons from *N* = 3 mice.

A recent study in hPSC‐derived sensory neurons demonstrated that although GIRK channels regulate neuronal excitability, their modulation by GABA_B_Rs was not observed, suggesting that functional GIRK1/2 channels were not coupled to GABA_B_Rs in these cells (St Clair‐Glover et al., 2025). However, this study identified a role for hyperpolarisation‐activated cyclic nucleotide‐gated (HCN) channels in regulating neuronal excitability. Specifically, application of the HCN inhibitor ZD7288 (30 µM) significantly altered both passive and active membrane properties in hPSC‐derived sensory neurons (St Clair‐Glover et al., 2025). Given these findings and our own observation that the GIRK inhibitor TPQ had no significant effect on excitability in colonic DRG neurons and afferents, we investigated whether HCN channels contribute to the regulation of excitability in mouse colonic DRG neurons. To test this, we applied 50 µM ZD7288 and assessed the electrophysiological properties of healthy mouse colonic DRG neurons using whole‐cell patch‐clamp recordings. Application of ZD7288 significantly increased neuronal excitability, as evidenced by a significant decrease in the amount of injected current required to evoke an action potential (*P* = 0.014) (Figure 7a, b). However, ZD7288 (50 µM) had no significant effect on the resting membrane potential (RMP; *P* = 0.398) of healthy mouse colon‐innervating DRG neurons (Figure 7c). Additionally, there were no significant differences in soma size between neurons that responded to ZD7288 (defined as >15% change in rheobase) and those that did not (*P* = 0.508) (*n* = 14, *N* = 3) (Figure 7d). Further analysis of action potential waveform characteristics revealed no differences in the ascending or descending phases of action potentials between neurons treated with ZD7288 and vehicle controls (Figure 7e, f).

## 4 DISCUSSION

GABA_B_Rs are widely expressed throughout the somatosensory nervous system, where they play a key role in modulating pain perception and transmission (Benke, 2022; Malcangio, 2018). Targeting these receptors has been shown to attenuate mechanical allodynia in neuropathic pain models (Zhang et al., 2022) and reduce colonic nociception, thereby alleviating visceral pain (Castro et al., 2017). As G protein‐coupled receptors, GABA_B_Rs mediate their inhibitory effects by modulating downstream ion channels (Alten et al., 2022; Qian et al., 2023). Notably, these downstream interactions are agonist‐specific, with distinct channel targets depending on the activating ligand (Shaye et al., 2021). For example, baclofen activation of GABA_B_Rs inhibits three distinct calcium channels, Ca_V_2.1, Ca_V_2.2, and Ca_V_2.3, whereas α‐conotoxin Vc1.1 selectively inhibits Ca_V_2.2 and Ca_V_2.3 channels, without affecting Ca_V_2.1 (Berecki et al., 2010). Additionally, GABA_B_R activation by Vc1.1 has been shown to activate GIRK channels in HEK293T cells co‐expressing either heteromeric human GIRK1/2 or homomeric GIRK2 subunits with GABA_B_Rs (Bony et al., 2022). This mechanism is particularly relevant given the widespread expression of GIRK channels throughout the central nervous system, including the spinal cord and sensory ganglia, where they regulate neuronal excitability and contribute to the pathophysiology of several neurological disorders (Luján & Aguado, 2015; Lüscher & Slesinger, 2010). GIRK channels hyperpolarize neurons in response to GPCR activation, modulating neuronal excitability through self‐inhibition, slow synaptic potentials, and volume transmission (Lüscher & Slesinger, 2010).

Activation of GIRK channels via GABA_B_Rs has been shown to modulate nociceptive transmission (Blednov et al., 2003; Ikeda et al., 2000; Mitrovic et al., 2003; Pan et al., 2008). In mouse DRGs, Vc1.1‐induced GABA_B_R activation potentiates GIRK channel currents, leading to membrane hyperpolarization and reduced neuronal excitability (Yousuf et al., 2022). In the present study, we characterized the expression profiles of Ca_V_ and GIRK channels, as well as GABA_B_R subunits, in both mouse and human whole DRG. A recent single‐cell nucleus RNA sequencing study reported high expression levels of Ca_V_ channels and GABA_B_Rs in human DRGs (Jung et al., 2023). Our findings align with that report, showing expression of GABA_B_Rs and Ca_V_s and their functional interactions. We also detected significant GIRK1 expression in human DRGs, with minimal or no expression of other GIRK isoforms, consistent with the single‐cell nucleus RNA sequencing data of individual human DRG neurons (Supporting information, Figure S1) (Jung et al., 2023).

**FIGURE 8:**
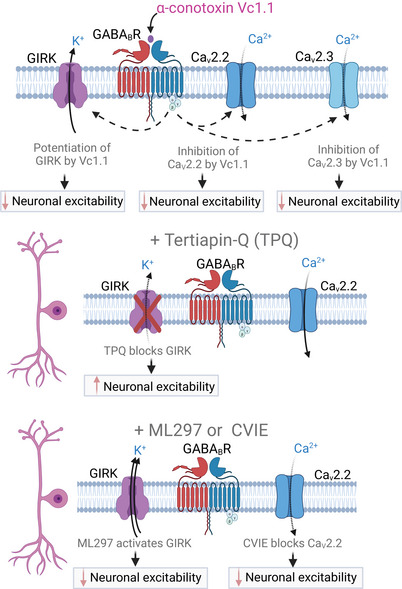
Summary of the mechanisms by which α‐conotoxin Vc1.1 inhibits mouse sensory neurons. Whole‐cell patch‐clamp recordings from mouse dorsal root ganglion (DRG) neurons show that α‐conotoxin Vc1.1 markedly reduces neuronal excitability by inhibiting Ca_V_2.2 and Ca_V_2.3 channels and by potentiating G protein‐coupled inwardly rectifying potassium (GIRK) channels. These findings highlight the dual mechanism of Vc1.1 in modulating sensory neuron activity. Inhibition of GIRK channels with the antagonist tertiapin‐Q (TPQ) increased neuronal excitability; however, this effect was not observed in DRG neurons innervating the mouse colon. In ‘general’ DRG neurons, inhibition of high voltage‐activated calcium (Ca_V_2.2) channels by the antagonist CVIE and activation of GIRK channels by the agonist ML297 both contribute to reduced neuronal excitability. Figure created with BioRender.

We further demonstrate that α‐conotoxin Vc1.1 inhibits neuronal excitability in mouse DRG through two distinct mechanisms: inhibition of HVA N‐type calcium (Ca_V_2.2) channels and potentiation of GIRK1/2 channels. Inhibiting GIRK1/2 channels enhanced neuronal excitability and attenuated the ability of Vc1.1 to suppress action potential firing. Collectively, these results confirm that α‐conotoxin Vc1.1 reduces neuronal excitability in mouse sensory DRGs via GABA_B_R‐mediated Ca_V_2.2 channel inhibition and GIRK channel potentiation (Figure 8) (Bony et al., 2022; Yousuf et al., 2022).

Our findings also provide important new insights into the downstream coupling of ion channels to GABA_B_Rs. Previous studies have shown that Vc1.1 enhances inwardly rectifying K^+^ currents in HEK293T cells expressing human GIRK1/2 channels and GABA_B_Rs (Bony et al., 2022). However, more recent studies using human pluripotent stem cell (hPSC)‐derived sensory neurons revealed that while GIRK channels modulate excitability, they do not appear to be functionally coupled to GABA_B_Rs in these cells (St Clair‐Glover et al., 2025). In the present study, we showed GIRK channels expressed in colon‐innervating DRG neurons, with GIRK1 being the most prevalent (58% of neurons), followed by GIRK2 (15%), whereas GIRK3 and GIRK4 were undetected. Notably, we also observed substantial overlap in the expression of GABA_B_R, Ca_V_2.2 and GIRK1 channels. However, our afferent nerve recordings and patch‐clamp studies revealed that GIRK channel inhibition had little effect on colonic afferent function in either healthy or CVH mice. This suggests that GIRK channels play a minimal role in regulating neuronal excitability of mouse colonic DRGs.

These findings contrast with previous studies showing that GIRK channels modulate neuronal excitability across general DRG populations. This discrepancy supports the view that colon‐innervating DRG neurons possess distinct functional and transcriptional profiles compared with DRG neurons innervating other tissues (Hockley et al., 2019; Hughes et al., 2007; Page et al., 2004). One possible explanation is that GABA_B_R–GIRK coupling is inactive or dormant under healthy conditions but becomes functional during inflammatory or pathological states. Similar state‐dependent receptor‐effector coupling has been reported for κ‐opioid and oxytocin receptors, which mediate anti‐nociceptive effects in colonic nociceptors only during inflammatory or post‐inflammatory CVH states, but not in healthy conditions (Brust et al., 2016; de Araujo et al., 2014; Hughes et al., 2014; Kremsmayr et al., 2024; Muratspahić et al., 2021). However, our patch‐clamp recordings from colon thoracolumbar DRG neurons in CVH states showed that GIRK channel inhibition with TPQ did not alter rheobase, further supporting a limited role for GIRK channels in high‐threshold nociceptors from the splanchnic pathway or in LT, WDR, or HT pelvic afferents in healthy states.

The analgesic effects of α‐conotoxin Vc1.1 on visceral pain are well established (Carstens et al., 2016; Castro et al., 2018, 2017; Sadeghi et al., 2018a). Vc1.1 activation of GABA_B_Rs inhibits Ca_V_2.2 and Ca_V_2.3 channels, thereby suppressing colonic DRG activity by increasing rheobase, reducing colonic afferent excitability, dampening nociceptive signalling in the spinal dorsal horn in response to colonic distension, and alleviating visceral pain in vivo (Castro et al., 2018, 2017; Sadeghi et al., 2018a). Notably, the anti‐nociceptive effects of Vc1.1 are enhanced in hyperalgesic states, as demonstrated in a CVH model, where upregulation of the Ca_V_2.2 exon 37a splice variant has been reported (Castro et al., 2017). While our data did not support a functional role for GIRK channels in colonic afferents, we identified a significant role for HCN channels in regulating colonic DRG neuronal excitability.

HCN channels are key regulators of neuronal excitability and have been implicated in both somatic (Emery et al., 2011) and visceral (Chen et al., 2014) pain. They are activated by membrane hyperpolarization, conduct both Na^+^ and K^+^ ions, and are modulated by cyclic AMP (cAMP), which facilitates channel opening (Benarroch, 2013; Porro et al., 2024). This is particularly relevant for GABA_B_R signalling, as cAMP‐dependent protein kinase phosphorylation can influence GABA_B_R‐effector coupling (Couve et al., 2002). Using the HCN channel inhibitor ZD7288, we demonstrated that inhibiting HCN channels altered neuronal excitability in healthy states without significantly affecting membrane potential or action potential properties. This finding is consistent with transcriptomic studies showing widespread expression of HCN1‐4 isoforms in colonic afferent DRG neurons (Supporting information, Figure S2) (Hockley et al., 2019). Further research is required to determine whether GABA_B_Rs directly interact with HCN channels in colonic DRGs and whether Vc1.1 activation of GABA_B_R modulates HCN channel activity in both healthy and disease states.

A key limitation of this study is the inclusion of samples from a single sex. Historically, many electrophysiological and neurophysiological studies have used male rodents exclusively to reduce variability associated with hormonal fluctuations in females (Tsao et al., 2023). While this approach enhances experimental consistency, it may limit the generalisability of the findings and overlook potential sex‐dependent differences in neuronal excitability or ion channel modulation, particularly since hormonal cycles in females can influence colonic sensitivity (Tramullas et al., 2021). Despite this limitation, our findings confirm that in mouse sensory DRG neurons, α‐conotoxin Vc1.1 reduces neuronal excitability downstream of GABA_B_R activation through both Ca_V_2.2 channel inhibition and GIRK channel potentiation. In contrast, in colonic‐innervating DRG neurons, despite robust GIRK1 expression, its functional contribution to afferent responses to mechanical distension or action potential generation was minimal. Instead, HCN channels appear to play a more prominent role in modulating excitability in these neurons, identifying them as promising targets for future investigation.

## AUTHOR CONTRIBUTIONS

SAHMRI: Mariana Brizuela: acquisition, analysis and interpretation of colonic afferent data and patch clamp data from colonic DRG neurons, drafting manuscript and figures, revisions for important intellectual content. Sonia Garcia‐Caraballo: acquisition, analysis and interpretation of QPCR from mouse and human DRG and single‐cell RT‐PCR from colonic DRG neurons, drafting manuscript and figures, revision for important intellectual content. Stuart M. Brierley: conceptualization of colonic studies, interpretation of data, project administration, funding acquisition, writing, review and editing, resources, methodology, supervision. University of Wollongong: Anuja R. Bony: acquisition, analysis and interpretation of patch clamp data from adult mouse DRG neurons, drafting manuscript and figures, revisions for important intellectual content. David J. Adams: conceptualization, interpretation of data, project administration, funding acquisition, writing, review and editing, resources, methodology, supervision. All authors approved the final version of the manuscript; agree to be accountable for all aspects of the work in ensuring that questions related to the accuracy or integrity of any part of the work are appropriately investigated and resolved; and all persons designated as authors qualify for authorship, and all those who qualify for authorship are listed.

## CONFLICT OF INTEREST

The authors have no competing interests in relation to the work contained within this study.

## Supporting information



Figure S1. Expression of GIRK, Ca_V_2.2, Ca_V_2.3, GABA_B_R1 and GABA_B_R2 from single cell transcriptomics in individual human DRG neurons.
Figure S2. Expression of HCN1–4 from single cell transcriptomics in individual mouse colon‐innervating DRG neurons.

## Data Availability

The data that support the findings of this study are available from the corresponding authors upon reasonable request.
