# Supplementary material for: GABAB receptor‐mediated modulation of sensory neuron excitability: Roles of CaV2.2, G‐protein‐coupled inwardly rectifying potassium (GIRK) channels, and hyperpolarisation‐activated cyclic nucleotide‐gated (HCN) channels in human and mouse nociception
Source: Exp Physiol. 2025 Nov 30;111(4):2026–43. doi: 10.1113/EP093318 (PMC13140288; doi:10.1113/EP093318)
Supplement: Supplementary file 1 — Figure S1. Expression of GIRK, CaV2.2, CaV2.3, GABABR1 and GABABR2 from single cell transcriptomics in individual human DRG neurons. Figure S2. Expression of HCN1–4 from single cell transcriptomics in individual mouse colon‐innervating DRG neurons. [file EPH-111-2026-s001.docx]

**Supplementary Information**

**GABA_B_ Receptor–Mediated Modulation of Sensory Neuron Excitability: Roles of Ca_V_2.2, GIRK, and HCN Channels in Human and Mouse Colonic Nociception**

**Running Title:** Ca_V_2.2, GIRK, and HCN channels in DRG neurons

Mariana Brizuela ^1^, Anuja R. Bony ^3^, Sonia Garcia Caraballo ^1^, David J. Adams ^3,*^, and Stuart M. Brierley ^1,2,*^

^1^ Visceral Pain Research Group, South Australian Health and Medical Research Institute (SAHMRI), North Terrace, Adelaide, SA 5000 Australia.

^2^ Faculty of Health and Medical Sciences, University of Adelaide, North Terrace, Adelaide, South Australia 5000, Australia.

^3^ Molecular Horizons/Faculty of Science, Medicine and Health, University of Wollongong, Wollongong, NSW 2522 Australia.

***Corresponding authors:**

[djadams@uow.edu.au](mailto:djadams@uow.edu.au) (<https://orcid.org/0000-0002-7030-2288>)

[stuart.brierley@sahmri.com](mailto:stuart.brierley@sahmri.com) (<https://orcid.org/0000-0002-2527-2905>)


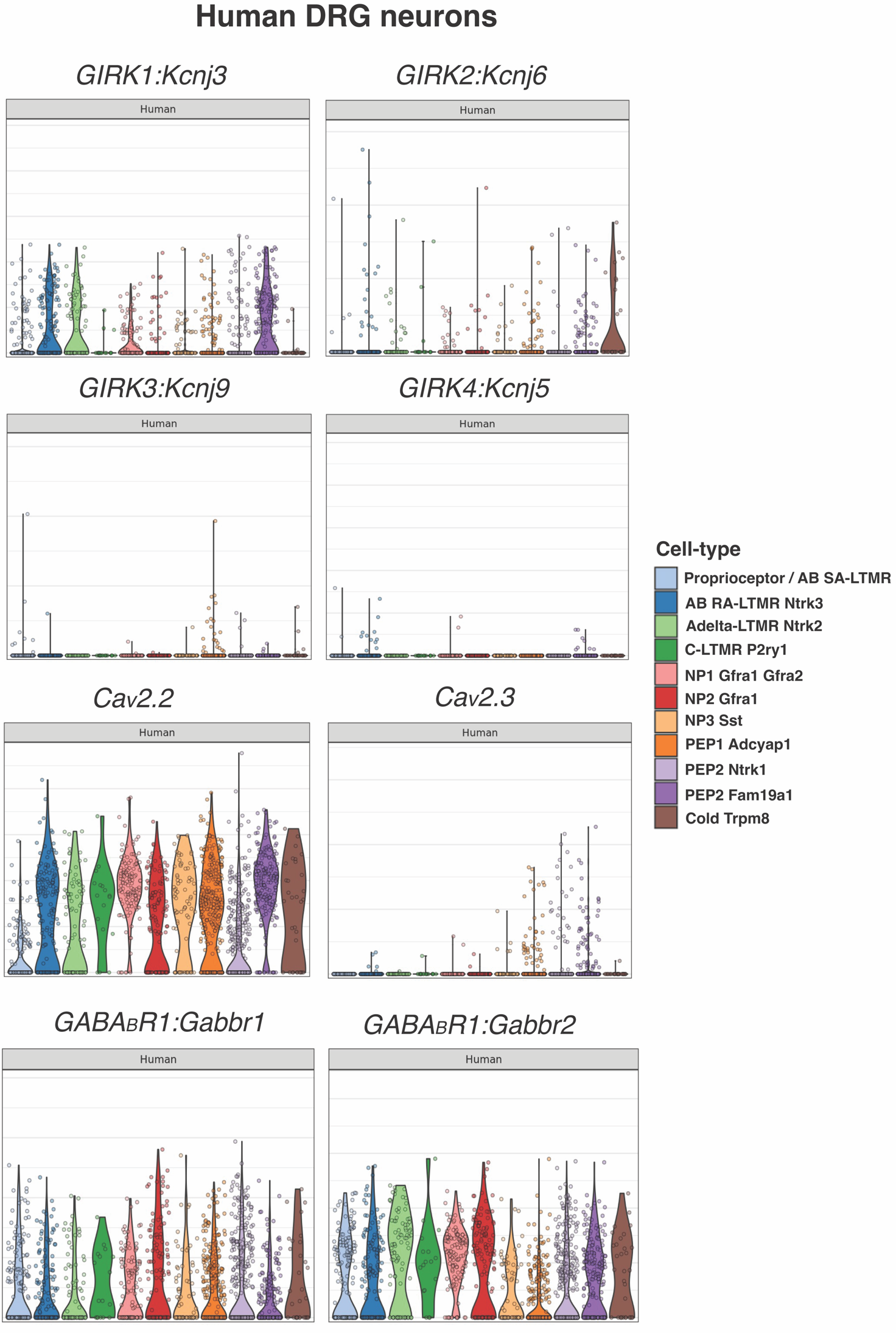


**Supplementary Figure 1. Expression of GIRK, Ca_V_2.2, Ca_V_2.3, GABA_B_R1 and GABA_B_R2 from single cell transcriptomics in individual human DRG neurons.**

Graphs were generated for comparative analysis by inputting the respective genes of interest into the XSpecies database (<http://research-pub.gene.com/XSpecies>), which compiles data generated from the study by Jung *et al.,* *“Cross-species transcriptomic atlas of dorsal root ganglia reveals species-specific programs for sensory function”.* (Jung *et al.*, 2023)


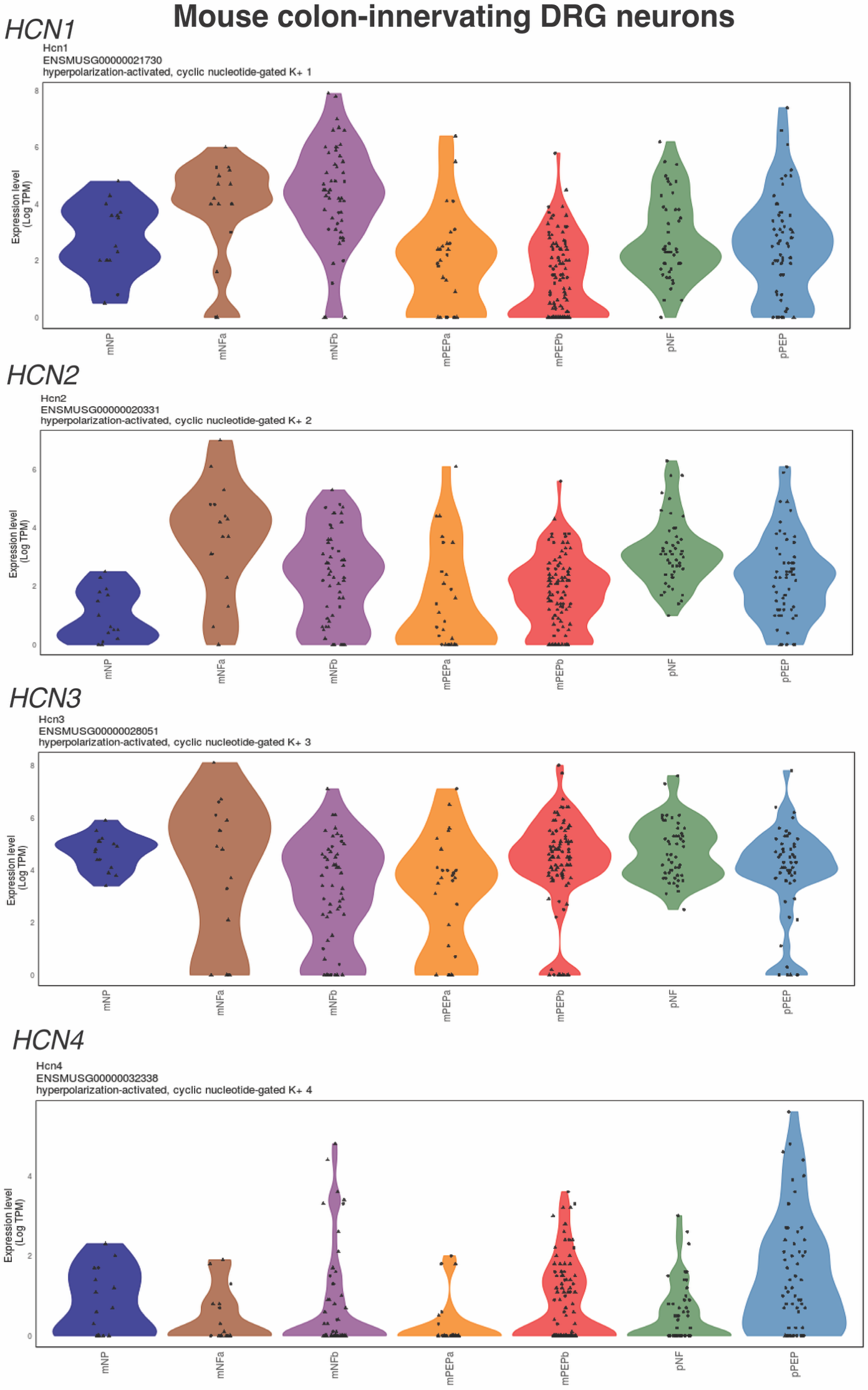


**Supplementary Figure 2. Expression of HCN1-4 from single cell transcriptomics in individual mouse colon-innervating DRG neurons.**

Graphs were generated for comparative analysis by inputting the respective genes of interest into the ColonicRNAseq database (<http://hockley.shinyapps.io/ColonicRNAseq>), which compiles data generated from the study by Hockley *et al.,* *“Single-Cell RNAseq Reveals Seven Classes of Colonic Sensory Neuron”.* (Hockley *et al.*, 2019)

**References**

Hockley, J. R. F., Taylor, T. S., Callejo, G., Wilbrey, A. L., Gutteridge, A., Bach, K., Winchester, W. J., Bulmer, D. C., McMurray, G., & Smith, E. S. J. (2019). Single-cell RNAseq reveals seven classes of colonic sensory neuron. *Gut*, 68(4), 633-644. <https://doi.org/10.1136/gutjnl-2017-315631>

Jung, M., Dourado, M., Maksymetz, J., Jacobson, A., Laufer, B. I., Baca, M., Foreman, O., Hackos, D. H., Riol-Blanco, L., & Kaminker, J. S. (2023). Cross-species transcriptomic atlas of dorsal root ganglia reveals species-specific programs for sensory function. *Nature Communications*, 14(1), 366. <https://doi.org/10.1038/s41467-023-36014-0>
